# Far-UVC light prevents MRSA infection of superficial wounds *in vivo*

**DOI:** 10.1371/journal.pone.0192053

**Published:** 2018-02-21

**Authors:** Brian Ponnaiya, Manuela Buonanno, David Welch, Igor Shuryak, Gerhard Randers-Pehrson, David J. Brenner

**Affiliations:** Center for Radiological Research, Department of Radiation Oncology, Columbia University Medical Center, New York, New York, United States of America; Georgetown University, UNITED STATES

## Abstract

**Background:**

Prevention of superficial surgical wound infections from drug-resistant bacteria such as methicillin resistant *Staphylococcus aureus* (MRSA) currently present major health care challenges. The majority of surgical site infections (SSI) are believed to be caused by airborne transmission of bacteria alighting onto the wound during surgical procedures. We have previously shown that far-ultraviolet C light in the wavelength range of 207–222 nm is significantly harmful to bacteria, but without damaging mammalian cells and tissues. It is important that the lamp be fitted with a filter to remove light emitted at wavelengths longer than 230 nm which are harmful.

**Aims:**

Using a hairless mouse model of infection of superficial wounds, here we tested the hypothesis that 222-nm light kills MRSA alighting onto a superficial skin incisions as efficiently as typical germicidal light (254 nm), but without inducing skin damage.

**Methods:**

To simulate the scenario wherein incisions are infected during surgical procedures as pathogens in the room alight on a wound, MRSA was spread on a defined area of the mouse dorsal skin; the infected skin was then exposed to UVC light (222 nm or 254 nm) followed by a superficial incision within the defined area, which was immediately sutured. Two and seven days post procedure, bactericidal efficacy was measured as MRSA colony formation unit (CFU) per gram of harvested skin whereas fixed samples were used to assess skin damage measured in terms of epidermal thickness and DNA photodamage.

**Results:**

In the circumstance of superficial incisions infected with bacteria alighting onto the wound, 222-nm light showed the same bactericidal properties of 254-nm light but without the associated skin damage.

**Conclusions:**

Being safe for patient and hospital staff, our results suggested that far-UVC light (222 nm) might be a convenient approach to prevent transmission of drug-resistant infectious agents in the clinical setting.

## Introduction

The majority of surgical site infection (SSI) are believed to be caused by airborne transmission of bacteria alighting onto the wound [1, 2]. Evidence for the dominance of the airborne route comes from correlations between the density of airborne bacteria and postoperative sepsis rates [2, 3]; proof for the impact of airborne bacteria alighting directly on the surgical wound comes, for example, from studies of conventional ultraviolet (UV) lamps specifically directed over the surgical site [1], and also from wound-directed filtered airflow studies [4].

Because of the prevalence of the airborne bacteria route, UV exposure during surgery has long been considered as a potential modality for reducing SSI. In fact there have been multiple clinical studies, starting as far back as 1940 [5, 6], demonstrating that UV exposure of the wound during surgery results in markedly decreased SSI rates.

SSI infections from drug-resistant bacteria such as methicillin resistant *Staphylococcus aureus* (MRSA) currently present a major health care burden. While significant resources have been applied to reduce their rates in the clinical setting, effective prevention remains elusive [7].

Surgical wound irradiation with conventional germicidal UV lamps typically emitting 254-nm light have shown great promise for infection control [1]: over a nineteen-year period following 5,980 joint replacements, the SSI rate using UV exposure decreased by three-fold (p< 0.0001), compared to unirradiated controls. These results, which are also consistent with direct measurement of bacterial loads in wounds with and without in-surgery UV exposure [8, 9] suggest that continuous UV exposure during surgery might reduce SSI rates significantly. However, the major downside of using conventional germicidal UV lamps during surgery is that exposure to germicidal 254-nm light is a health hazard, causing skin cancer and cataracts [10, 11]. This necessitates the use of cumbersome personal protective equipment for patient and hospital staff, which has prevented widespread adoption of germicidal UV lamps during surgical procedures.

Previously we have shown that far-UVC light in the range of 207–222 nm kills bacteria efficiently, but without the skin damaging effects associated with conventional germicidal UV exposure [12–14]. Another advantage of the ability of far-UVC light to selectively inactivate microorganisms while preserving the viability of mammalian host cells and tissues, is promotion of wound healing and skin homeostasis [9, 15, 16]. In fostering fibronectin production, UVC stimulates cell migration and the production of growth factors [17], which further augment the healing cascade.

Based on our earlier studies *in vitro* and *in vivo*, here we hypothesized that exposure of far-UVC light onto the surgical wound area during surgery is a suitable approach to killing bacteria both in the air and as they lay down onto the wound area or settle on the surgeon's hands and instruments, without adverse health hazards for patient or staff [18, 19].

To mimic the scenario wherein incisions are infected during surgical procedures as bacteria in the room alight on the skin, we developed a mouse protocol in which MRSA was spread on a defined area of the skin and then exposed to UVC light [20]. We measured bactericidal efficacy (CFU/g) and effects on skin (i.e. epidermal thickness and DNA photodamage) two and seven days after exposure to different fluences of 222-nm light vs. 254-nm light.

## Material and methods

### UV lamps

We used an excimer USHIO (Japan) lamp prototype based on a krypton-chlorine (Kr-Cl) gas mixture that emits principally at 222 nm. The lamp was air cooled and light exited the lamp from a 60 cm^2^ exit window. A built in filter was used to remove essentially all but the dominant 222-nm wavelength emission. A study using an unfiltered 222-nm lamp found significant damage to the skin of human volunteers at doses at the low end of those used here [21]. A UV spectrometer (Photon Control, BC, Canada) sensitive in the wavelength range from 200–360 nm was used to characterize the wavelength spectra emitted by the USHIO lamp, and a deuterium lamp standard with a NIST-traceable spectral irradiance (Newport Corp, Stratford, CT) was used to calibrate the UV spectrometer.

Studies were also carried out with a low-pressure mercury germicidal lamp (Thera-Wand^™^ C-100, Biomation Almonte, ON Canada) with peak emission at 254 nm and used as positive control. An UIT-250 UV meter (Ushio, Cypress, CA) was used to measure the fluence rate from both lamps. Samples were positioned 32 mm away from the 222-nm Ushio lamp at which the power density was 5 mW/cm^2^. The Thera-Wand instead was positioned 55 mm from the mouse at which corresponded a power density of 3.5 mW/cm^2^. Mice were exposed to 40 or 300 mJ/cm^2^.

### MRSA culture

Fresh colonies of methicillin resistant *S*. *aureus* (MRSA USA300, multilocus sequence type 8, clonal complex 8, staphylococcal cassette chromosome mec type IV) were kindly provided by Dr. Frank D. Lowy. Firstly reported as cause of skin and soft issue infection, USA300 isolates have quickly developed resistance to antimicrobial agents thereby becoming the cause of community- and hospital-acquired invasive diseases such as bacteraemia, endocarditis, and pneumonia [22]. The day before the experiment, MRSA was grown overnight at 37°C into tryptic soy broth (Thermo Scientific, Pittsburgh, PA, USA). The culture was then resuspended in fresh broth and grown to mid-log phase for 2 h. Bacteria were collected by centrifugation, washed, resuspended in broth, and adjusted to an optical density at 600 nm of 0.5. Dilutions were performed in HBSS (Hanks’ balanced salt solution) to yield to a bacterial concentration of 10^9^ CFU/ml.

### Mouse skin infection and irradiation

Six- to eight-week-old male hairless mice (SKH1-Elite strain 477, Charles River Laboratories, Stone Ridge, NY) were divided in two groups. A total of 100 mice in Group 1 was used to establish that i) 25 μl of 10^9^ CFU/ml MRSA was the concentration that resulted in > 90% wound infection rate and ii) 40 and 300 mJ/cm^2^ were the optimal fluences required to prevent infections from either the 222-nm or 254-nm light. Group 2 consisted of a total of 36 animals, 16 of which were sacrificed two days after UV exposure and 20 after seven days, corresponding to at least three animals per treatment / time point.

Mice were anesthetized via isoflurane inhalation (4% with 2L / min O_2_), with subcutaneous injections of the local anesthetic bupivicaine (2 mg/kg) and the analgesic carpofen (5 mg/kg). Carpofen (5 mg/kg) was also injected 24 h after UV exposure. After anesthesia, an iodine povacrylex solution (Duraprep, 3M, St. Paul, MN) was applied on the dorsal skin and let dry for 5 minutes. In addition to sterilizing the surgical site, Duraprep forms a water-insoluble film thereby preventing bacteria applied subsequently on the skin from getting into the skin pores and becoming inaccessible to the action of UV light. The sterilized dorsal surface of each mouse was covered with an adhesive surgical drape containing povidine, with a 5 cm x 4 cm window cut out. A 1 cm x 2 cm region was marked out on which 25 μl of 10^9^ CFU/ml MRSA were spread with a low retention swab. Following 5 minute to let the MRSA solution to dry, the infected skin was exposed to 40 or 300 mJ/cm^2^ from the 222-nm or the 254-nm emitting lamp. After UV exposure, an incision was made in the region and immediately sutured. Negative controls consisted of mice whose incision was uninfected by applying 25 μl of saline prior to exposure to either UV lamp; positive controls consisted of mice whose incision was infected but not UVC-irradiated.

Two or seven days after surgery, the mice were anesthetized via isoflurane inhalation (4% with 2L / min O_2_) and the skin was harvested for analysis. The mice were subsequently humanely euthanized by CO_2_ inhalation followed by cervical dislocation, in accordance with federal guidelines and protocols approved by the Columbia University Medical Center IACUC.

Skin can be infected before obvious signs of infection such as redness and pus appear. Therefore, a skin sample was classified as infected if it yielded to colonies assessed by the colony formation unit (CFU) assay described below. For each treatment, the percentages of mice whose skin yielded to colonies measured with the CFU assay divided by the total number of samples are reported in Table 1.

**Table 1:** Percentage of infected mouse skin samples 2 or 7 days after UVC exposure. For each treatment, the value represents the percentage of mice whose skin yielded to colonies measured with the CFU assay divided by the total number of samples.

Sample	% infected skin samples	
	Day 2	Day 7
Saline (negative control)	0.0	0.0
40 mJ/cm^2^–222 nm	100.0	50.0
40 mJ/cm^2^–254 nm	66.7	50.0
300 mJ/cm^2^–222 nm	66.7	0.0
300 mJ/cm^2^–254 nm	33.4	33.4
MRSA (positive control)	100.0	100.0

### Bacterial counts

Survival of MRSA as a function of UVC fluence was assessed with the standard colony forming unit (CFU) assay, as previously described (7). Briefly, the skin was weighed and then homogenized in 0.9 ml of HBSS containing three 3.2-mm beads (SSB32, Next Advance, Rochester, New York) using a Bullet Blender Storm (BBY24M, Next Advance) for 5 minutes at the maximum speed. 50 μl of serial dilutions of each homogenate were plated on mannitol salt agar plates (BD Diagnostic System, Sparks, MD) and incubated for 48 h at 37°C for colony formation. Data are presented in CFU/g.

### Mouse skin safety-specific endpoints

To assess skin damage, tissue samples were harvested two or seven days after UV exposure and fixed overnight in 10% formalin. Samples were paraffin-embedded and stained with hematoxylin and eosin to measure epidermal thickness; yields of UV-induced DNA photodamage were assessed using the immunohistochemical protocol previously described [12, 14]. Tissues were examined with an Olympus IX70 microscope equipped with a Photometrics^®^ PVCAM high-resolution, high-efficiency digital camera and Image-Pro Plus 6.0 software was used to analyze the images. For each mouse, each endpoint was measured in at least six randomly selected fields of view.

### Statistical analysis

Comparisons of mean values between treatment groups and controls were performed using Student's t test and comparison of proportions were assessed with standard χ^2^ tests. Data were analyzed using multiple linear regression, separately for bactericidal efficacy and skin safety endpoints. The dependent variables were i) the log_10_-transformed concentration of MRSA colony forming units per gram (Table 2), ii) Epidermal thickness (Table 3), and iii) cyclobutane pyrimidine dimers (CPDs) (Table 4). Several independent (predictor) variables including UV type (either 222 nm or 254 nm), fluence (mJ/cm^2^), and time (days) were used in each analysis. The best-fit model coefficients, standard errors and p-values are reported (Tables 2–4). The log_10_ transformation of CFU/g data was done to bring the error distribution closer to normal. Models containing all possible predictor combinations and 2-way interactions between them were fitted to the data and ranked by Akaike information criterion with sample size correction (AICc) using the *glmulti* package in R software (version 3.2.3). Importance scores were calculated for each predictor and predictor combination based on Akaike weights, using the same software. Only those predictors and predictor interactions that achieved the highest importance scores were retained in the selected model.

**Table 2:** Multiple linear regression for the dependent variable log_10_-transformed concentration of MRSA colony forming units per gram, log_10_. The variable UV type indicates 254 nm light, as compared with 222 nm light.

Predictor	Best-fit coefficient	Standard error	p-value
Intercept	7.550	0.645	3.91×10^−10^
UV type (254 nm)	-0.337	0.513	0.519
Fluence (mJ/cm^2^)	-5.915×10^−3^	1.898×10^−3^	5.69×10^−3^
Days	-0.579	0.103	2.09×10^−5^

**Table 3:** Multiple linear regression with the dependent variable epidermal thickness (μm). The variable UV type indicates 254 nm light, as compared with 222 nm light.

Predictor	Best-fit coefficient	Standard error	p-value
Intercept	3.546	0.935	8.40×10^−4^
UV type (254 nm)	0.312	0.914	0.736
Fluence (mJ/cm^2^)	3.375×10^−3^	4.601×10^−3^	0.470
Days	0.046	0.145	0.752
Dose*Days	-1.222×10^−3^	0.732×10^−3^	0.107
Dose*UV type (254 nm)	0.011	0.004	0.013

**Table 4:** Multiple linear regression with the dependent variable proportion of keratinocytes with CPDs. The variable UV type indicates 254 nm light, as compared with 222 nm light.

Predictor	Best-fit coefficient	Standard error	p-value
Intercept	-3.794	0.857	9.62×10^−6^
UV type (254 nm)	2.711	0.840	1.24×10^−3^
Fluence (mJ/cm^2^)	6.629×10^−3^	4.033×10^−3^	0.100
Days	-0.101	0.049	0.042
Dose*Days	-2.014×10^−3^	0.382×10^−3^	1.37×10^−7^
Dose*UV type (254 nm)	9.848×10^−3^	4.196×10^−3^	0.019

## Results

### Bactericidal efficacy

**Fig 1:**
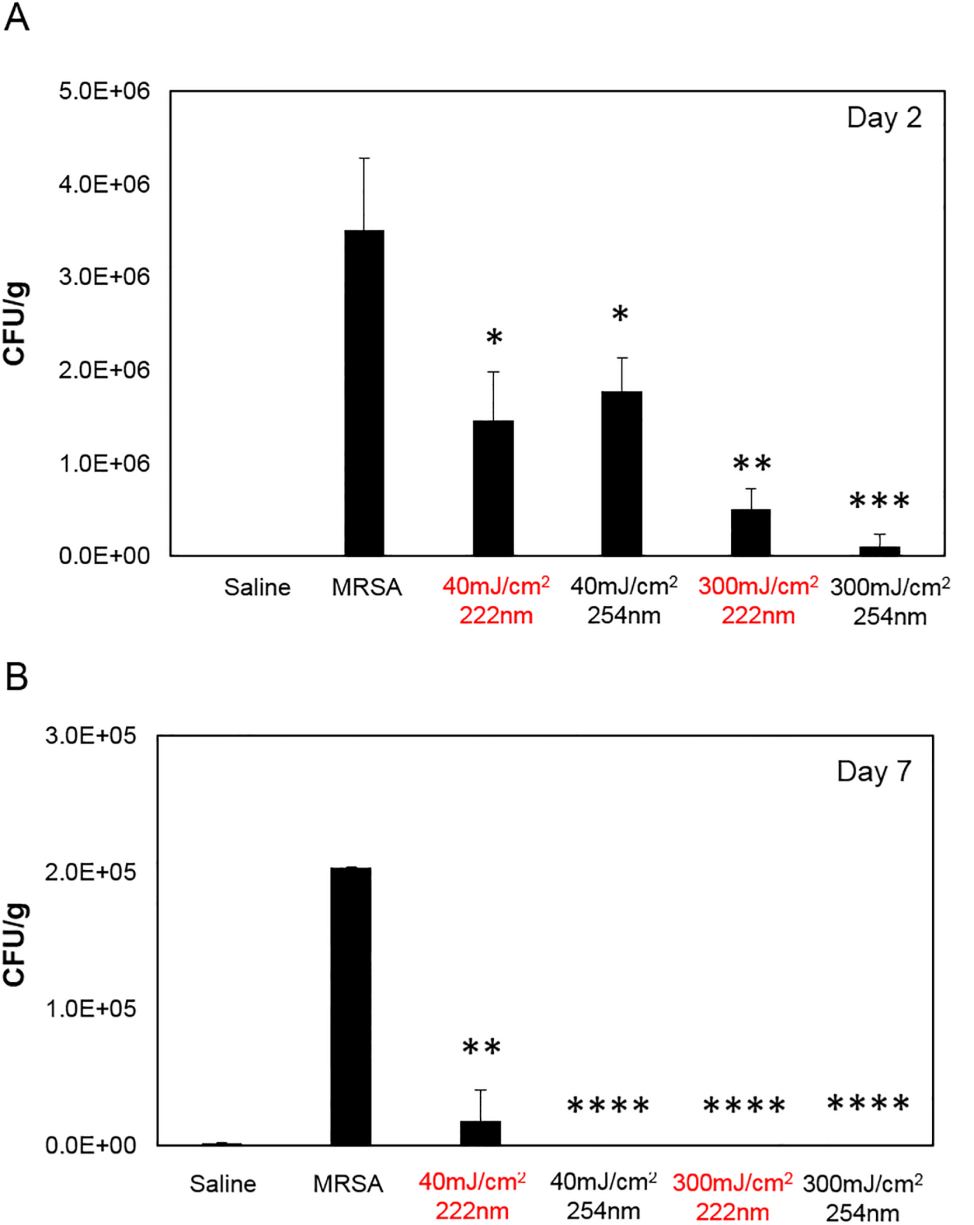
Bactericidal efficacy of UVC light of superficial wounds infected with MRSA. CFU/g of mouse skin where MRSA was spread before exposure to 40 or 300 mJ/cm^2^ delivered by the 222- or 254-nm light, and followed by a superficial incision. Negative controls were obtained from skin incisions that were uninfected by applying saline prior to exposure to either UV lamp (Saline), whereas skin incisions that were infected but not exposed to the UV light (MRSA) represented the positive controls. Tissues were harvested at A) day 2 or B) day 7 after exposure. *p< 0.05, **p< 0.01, ***p< 0.005, ****p< 0.0001 compared to positive controls (MRSA).

Fig 1 shows bacteria counts (CFU/g) in skin of mice two days (Fig 1A) or seven days (Fig 1B) after infection with MRSA and subsequently exposed to 40 or 300 mJ/cm^2^ from 222- or 254-nm light. The results were compared with bacterial counts measured in skin incisions that were uninfected by applying saline prior to exposure to the UV light (sample denoted as Saline) and to those obtained from skin incisions that were infected but not UVC-irradiated (sample denoted as MRSA).

Compared to incisions that were infected but not UVC-irradiated (MRSA), both fluences from either 222- or 254-nm light resulted in a statistically significant reduction of bacteria counts on day 2 day 7 (see Fig 1 caption for specific p values). Importantly, 222-nm light appeared to be as effective as the 254-nm light in killing MRSA. Specifically, multiple linear regression analysis (Table 2) indicated that i) bacterial CFU concentration had a statistically significant negative dependences on UV dose (p = 5.69×10^−3^) and time (p = 2.09×10^−5^) suggesting that both 222- and 254-nm lights were effective against MRSA and significantly reduced MRSA numbers compared with unirradiated controls; ii) there was no significant difference between UV types (222 nm vs. 254 nm) (p = 0.519) indicating that both wavelengths were similarly effective in killing MRSA.

### Skin safety

**Fig 2:**
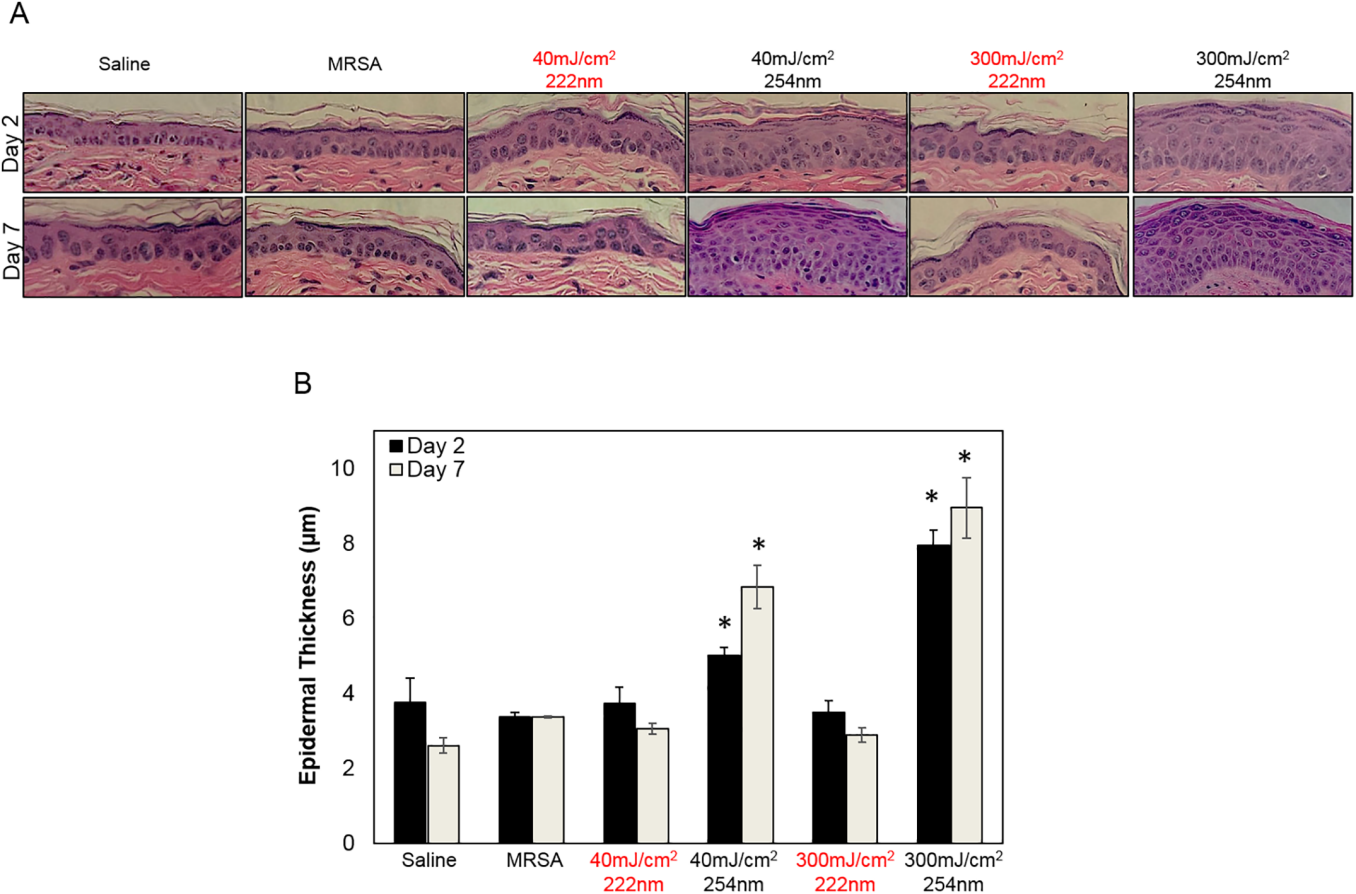
Epidermal thickness of mice skin infected with MRSA and exposed to UVC light. A) Representative cross-sectional images of hematoxylin and eosin-stained mouse dorsal skin comparing the epidermal thickness two or seven days after exposure to 40 or 300 mJ/cm^2^ from the 222-nm or 254-nm light to that of skin incisions that were uninfected by applying saline prior to exposure to either UV lamp (Saline). Skin incisions that were infected but not exposed to the UV light (MRSA) represented the positive controls. B) Quantification of epidermal thickness; values represent the average ± SD measured in nine randomly selected field of view per mouse. *p< 0.05, **p< 0.005 compared to positive controls (MRSA).

**Fig 3:**
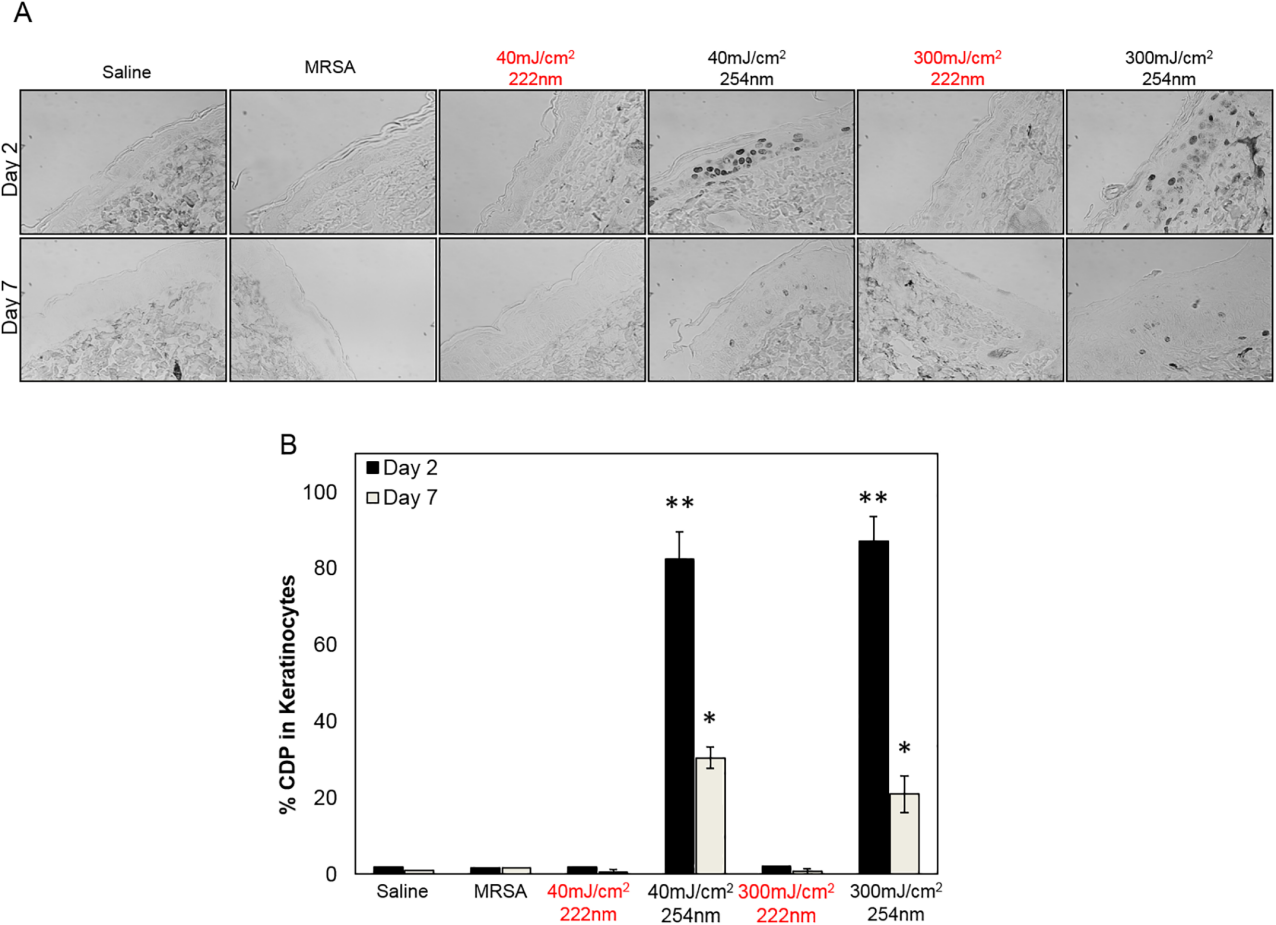
DNA photodamage in mice skin infected with MRSA and exposed to UVC light. A) Representative cross-sectional images of dorsal skin comparing the percentage of pre-mutagenic skin lesions CPD in keratinocytes (dark-stained cells) two or seven days after exposure to 40 or 300 mJ/cm^2^ from the 222-nm or 254-nm light to that of skin incisions that were uninfected by applying saline prior to exposure to either UV lamp (Saline). Skin incisions that were infected but not exposed to the UV light (MRSA) represented the positive controls; B) Quantification of keratinocytes showing CPD; values represent the average ± SD measured in nine randomly selected field of view per mouse. *p< 0.05, **p< 0.005 compared to positive controls (MRSA).

We measured epidermal thickness and DNA photodamage as markers of skin damage two and seven days after UVC exposure (Figs 2 and 3). Unlike exposure to the 254-nm light (p< 0.05) [12, 19], skin of mice exposed to low or high fluences from the 222-nm light did not induce a statistically significant increase in epidermal thickness compared to control (Saline) at neither time point after exposure (Fig 2).

Using the statistical methods described above and reported in Table 3, the analysis suggests that compared to unirradiated controls (Saline), 222-nm UV had no statistically significant effect on mean epidermal thickness (p = 0.47); in contrast, high doses of 254-nm UV were associated with increased mean epidermal thickness (p = 0.013).

Fig 3 shows DNA photodamage as percentage of cyclobutane pyrimidine dimers (CPD) induced by 40 or 300 mJ/cm^2^ from the 222-nm lamp or the 254-nm emitting lamp, two or seven days after exposure. In agreement with our previous studies [12, 14, 19], 254-nm light produced high yields of CPD at both fluences that were significantly higher than controls (p< 0.005) and persisted up to seven days after exposure (p< 0.05). In contrast, neither fluence from 222-nm light produced the pre-mutagenic skin lesions.

Multiple linear regressions conducted on the CPDs data (Table 4) indicated that compared with unirradiated controls i) 254 nm UV significantly increased the proportion of cells with CPDs, both in terms of a main effect (p = 1.24×10^−3^) and an interaction with dose (p = 0.019). In contrast, 222-nm light did not significantly increase the proportion of cells with CPDs above control levels (p = 0.1); ii) the significant negative dependences of the proportion of cells with CPDs on Days and Dose*Days may represent repair of these lesions after irradiation.

## Discussion

The incidence of surgical site infections is on the increase worldwide [23]. Although occurrence of SSI depends on both patient- and procedure-specific factors, it has been estimated that up to 90% of pathogenic bacteria detected from surgical wounds were related to airborne particles in the operating room [24]. During surgical procedures, bacteria-laden airborne particles may directly enter the surgical site or settle on surgical instruments, resulting in SSI [25].

Airborne transmission is known to be the infection route for diseases such as tuberculosis and it has also been implicated in nosocomial infections involving drug-resistant bacteria such as MRSA [26]. Although several systems for air decontamination in the operative rooms have been devised with different degrees of success [27, 28], other approaches are needed to adequately prevent SSI. In fact, since 2007 the Centers for Disease Control and Prevention (CDC) have declared the prevention and control of multidrug-resistant organisms a national priority [29].

The germicidal effectiveness of ultraviolet (UV) light for disinfection of airborne pathogens is well-known [1]. Recently, irradiation of circulating room air with UV, also known as upper-room ultraviolet germicidal irradiation (UVGI), has shown considerable promise [30, 31]. However, its widespread use in public settings has been halted by the fact that conventional UVC light sources, typically emitting at 254 nm, are a human health hazard causing skin cancer and cataracts [10, 11].

In contrast, we have previously shown that far-UCV light in the range of 207–222 nm has the same bactericidal potential of 254-nm light, but without the damaging effects to mammalian cells and tissues [13, 18]. The biophysical explanation is based on the strong absorbance of far-UVC light by cellular proteins; moreover, due to its short range in biological materials, far-UVC light cannot penetrate the outer layer of the skin as well as the outer surface of the eyes. In contrast, because microbes are typically of micron or smaller dimensions, far-UVC light can efficiently inactivate their nuclei acids thereby impairing the microbes’ proliferative capacity [13].

Here we tested the hypothesis that far-UVC light (i.e. 222 nm) can efficiently prevent MRSA infection in a hairless mouse model of superficial skin incisions. To this aim, we have developed a protocol to simulate superficial wounds infected with bacteria potentially alighting onto the surgical wound from the room air or that for instance may be carried by bacteria settling on the clinical staff hands or surgical tools. Via multiple linear regression analysis, we showed that 222-nm light had the same bactericidal properties of 254-nm light (Fig 1 and Table 2), but without causing skin damage, up to seven days after exposure (Figs 2 and 3). In particular, 254-nm light induced a statistical significant increase in pre-malignant DNA lesions (CPDs) in epidermal keratinocytes (p = 1.24×10^−3^), which persisted for up to seven days after exposure. In agreement with our previous results [13, 14, 19] however, 222-nm light did not significantly increase the proportion of cells with CPD above control levels (p = 0.1) (Fig 3B and Table 4).

Several recommendations from the CDC to prevent intraoperative infections including air ventilation and cleaning and disinfection of environmental surfaces are routinely followed in US hospitals [32, 33]. Nevertheless, 77% of the deaths following complications from surgery were reported to be related to SSI [33]. Recent studies have revealed that a large number of airborne particles are produced during typical actions by the surgical staff such as unfolding the surgical gown, removal of gloves and placing arms through the sleeves of the gowns [20]. Therefore, it can be envisioned that continuous use of far-UVC light during surgical procedures might be an advantageous approach to inactivate bacteria as they alight on the wound [1], thus preventing infection as well as the formations of bacterial clusters or biofilms. In conclusion, being safe for patient and staff, far-UVC light can be implemented as one of the standard precautions to prevent transmission of infectious agents in the clinical setting.
